# Supplementary material for: Neutral particle mass spectrometry with nanomechanical systems
Source: Nat Commun. 2015 Mar 10;6:6482. doi: 10.1038/ncomms7482 (PMC4366497; doi:10.1038/ncomms7482)
Supplement: Supplementary Information — Supplementary Figures 1-7, Supplementary Methods and Supplementary References [file ncomms7482-s1.pdf]

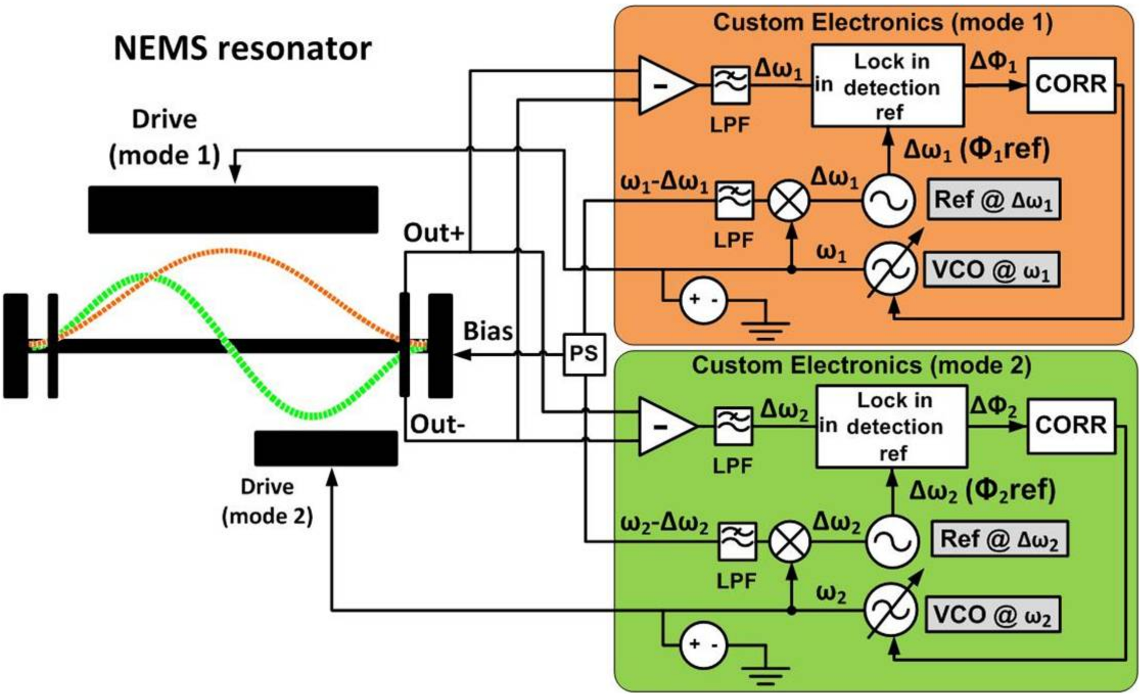

Supplementary Figure 1: Diagram of the NEMS resonator and its associated custom electronics to simultaneously track mode 1 and mode 2 resonances with PLLs. Two separate electronic boards, each assigned to a given resonance mode, are combined with a Power Splitter (PS) for nanogauge polarization and drive their assigned mode with the appropriate capacitive electrode. For each mode the beam is driven at ω (some tens of MHz) and the bias frequency is at ω – Δω to downmix the output at Δω (some tens of kHz) and circumvent signal attenuation caused by parasitic impedances. The resonator outputs are distributed to the differential inputs of both electronic boards and readout by Lock-In Amplifiers (LIA). In Closed Loop operation, the output signal phase difference is shaped by a corrector (CORR) that feeds a Voltage Controlled Oscillator (VCO) driving the NEMS resonator at its resonance frequency. The corrector is a robust H∞ controller whose coefficients are calculated using a loop shaping method[1].

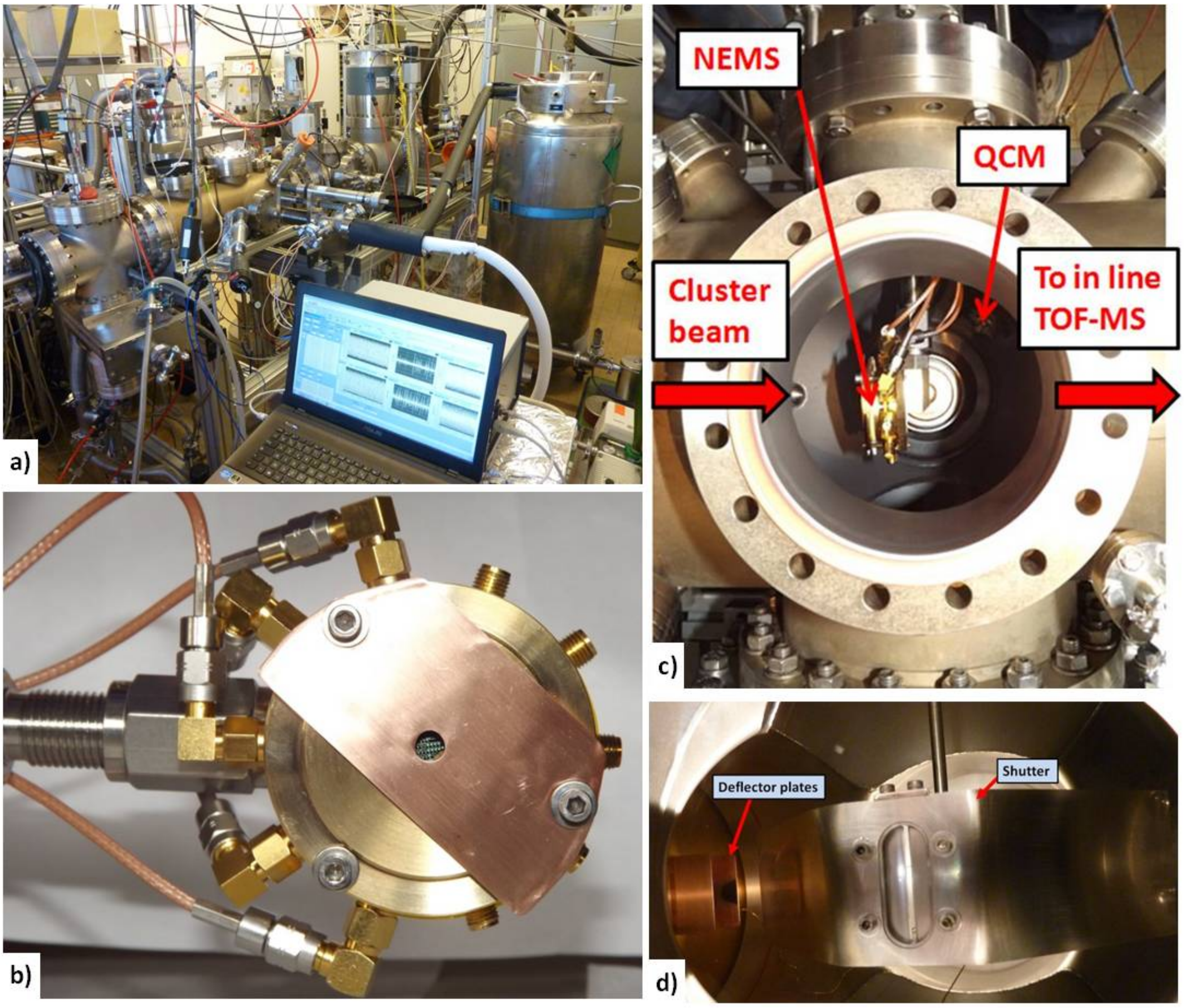

Supplementary Figure 2: Hybrid NEMS and TOF-MS setup. General view of the complete setup (a) and front view of the NEMS holder engineered to provide electrical connections and thermal contact (b). A protection hood (here a copper sheet) is mounted to localize cluster deposition on the wirebonded device, keeping most of the other devices and the PCB intact for subsequent experiments. (c) shows a top view of the deposition chamber: coming from the intermediate chamber, the clusters can either hit the NEMS or the QCM set on retractable arms or enter the in-line TOF mass spectrometer. In the deflection chamber, deflector plates may be used to remove ionized particles (d). A shutter, not used in this study, may be used to reduce the particle flux.

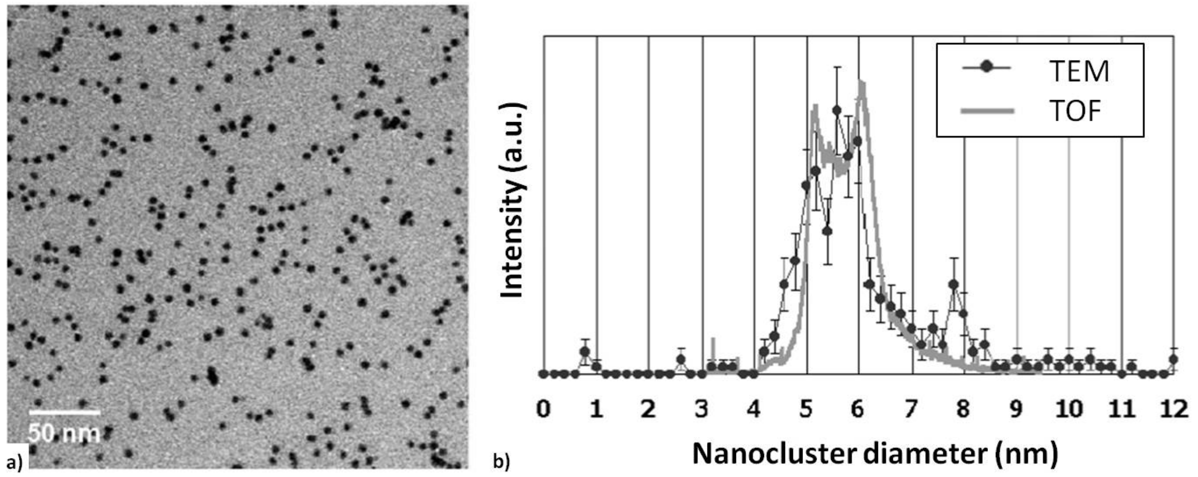

Supplementary Figure 3: Confirmation of TOF calibration in the high mass range. A population of Cobalt nanoclusters, previously characterized by TOF mass spectrometry, is deposited on a microscope grid covered by a thin carbon sheet and observed by TEM. The resulting image (a) is analyzed by a numerical algorithm which bestows (x, y) coordinates and a pixel number for each cluster. Knowing the image scale, and assuming that clusters are spherical, the size (or mass) distribution of the cluster population can be determined and compared to the TOF spectra (b). A good matching is observed between the two populations, except for one peak at 7.8nm. This is an artifact induced by the image processing algorithm where two clusters too close to each other are considered as one.

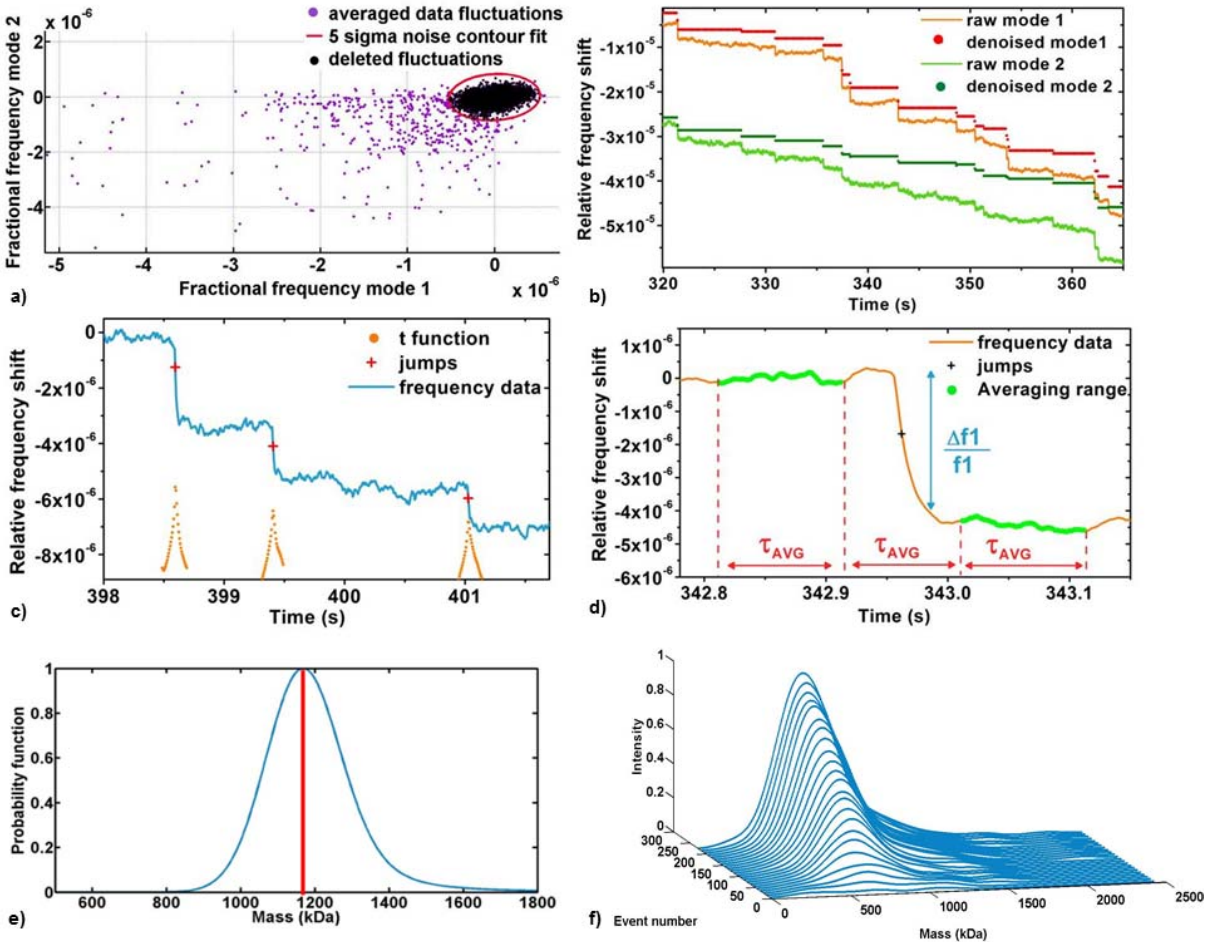

Supplementary Figure 4: Jump detection algorithm. Fractional frequency shifts of mode 2 versus relative frequency shifts of mode 2 are represented and the resonator noise is removed thanks to a 5-sigma noise contour fit obtained from previous acquisitions without particle accretion (a). (b) shows the effect of denoising on mode 1 and mode 2 relative frequencies. Mass induced frequency jumps are evidenced. The center of the frequency jumps is found with the maximum of the Student's t-test (c). The *t* function is computed only over ranges where events occur. The frequency jumps are then evaluated by subtraction of averaged values before and after the jump (d). e) A pair of frequency shifts $[\mathbf{\delta f_1}, \mathbf{\delta f_2}]$ is translated into a mass/position pair $[\boldsymbol{\delta m}, \boldsymbol{x}]$. The central mass $\boldsymbol{\delta m}$ is which of the maximum occurrence probability, in red. The uncertainty on the deduced central mass inferred from the measured frequency fluctuations is represented in blue. f) The spectrum is constructed by summing all individual events.

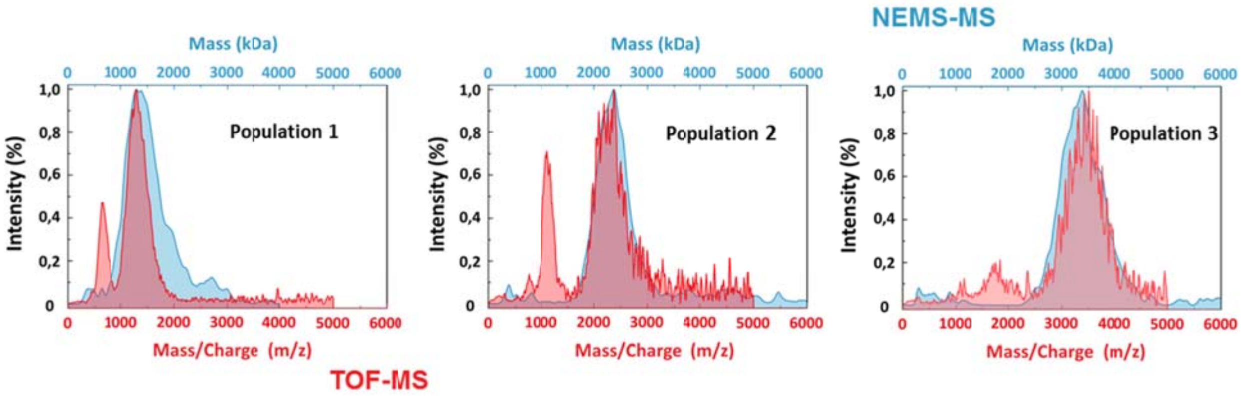

Supplementary Figure 5: TOF-MS and NEMS-MS spectra superimposed for three different tantalum cluster populations.

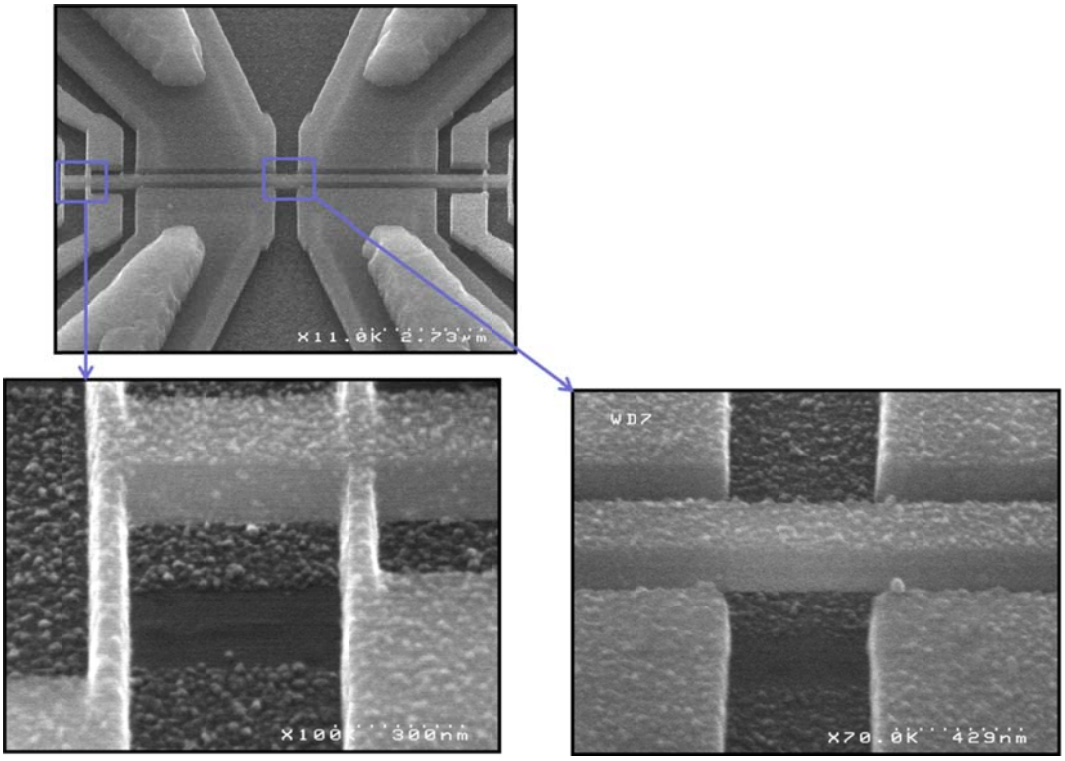

Supplementary Figure 6: Uniform deposition of small copper clusters with high flux on the resonator surface. The clusters can be clearly seen on the resonator as well as on the silicon substrate beneath, except where the resonator shadowed the deposition.

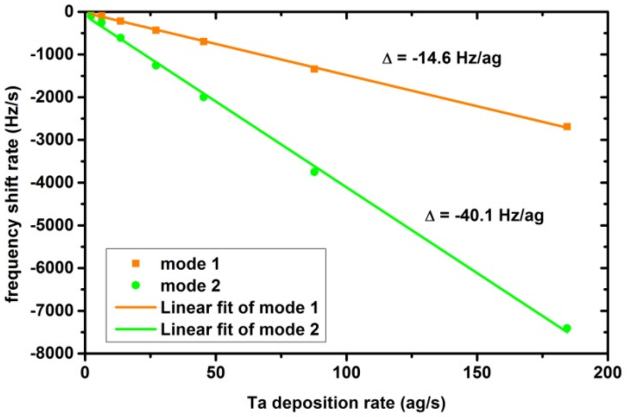

Supplementary Figure 7: Sensitivity calibration method: frequency responses to five different deposition rates measured by the QCM are acquired. The NEMS frequency shift rates versus the mass deposition rate on the device deduced from the QCM are linearly fitted and plotted to obtain the experimental mass sensitivities.

Supplementary Methods

Silicon resonator fabrication

The resonant NEMS fabrication steps use state-of-the-art microelectronics fabrication processes following a top-down approach on a 200 mm silicon-on-insulator (SOI) of (100) orientation with a p-doped top silicon layer (~5×10^{19} cm^{-3}). The top silicon layer is intended to form the resonant area as well as provide electrical access, while the buried oxide is used as a sacrificial layer eventually released by vapor HF. The resonant beam is patterned by a hybrid deep UV/electron beam lithography technique. AlSi metal lines are deposited on the existing Si contacts to minimize electrical access resistance and form metallic pads suitable for wirebonding. The resonator beam is released by vapor HF. Details can be found elsewhere[2].

Electrical Readout

The electronics schematic is described Supplementary Figure 1: The readout of each mode is performed thanks to a downmixing scheme[3]. Real-time mass sensing of an individual particle requires the closed-loop operation of at least two modes. The frequency measurement of the two first modes is performed simultaneously in a similar fashion as in [4]. Closed-loop operation is performed thanks to a Phase Lock Loop (PLL). During the data acquisition, a fast response time (denoted hereafter τ_{PLL}) of 10ms (as the 90th percentile) was used to handle the high particle landing rate on the resonator. The data was averaged on 3 times this timescale to ensure an accurate response of the loop (see Supplementary Methods, section “Jump Detection Algorithm” for more details). We typically measured an Allan deviation[2] of 10^{-7} for both modes, yielding a theoretical mass resolution of ~70 kDa.

Nanocluster growth & detection

The cluster growth is performed by sputtering a metallic target with a magnetron head inserted in a chamber cooled with liquid nitrogen. Argon is chosen as the sputtering gas, cooling gas and carrier gas due to its high atomic number and low chemical reactivity. Positive argon ions generated in the plasma bombard at high speed a metallic target polarized at about –150 V, ejecting metallic atoms and secondary electrons that further maintain the plasma. The permanent magnet traps the secondary electrons in helical paths around the magnetic lines hence confining the plasma over to the target surface. This results in higher ionizing collision rates with argon leading to higher sputter rates. By colliding with the cold argon gas, the sputtered atoms aggregate in the growth zone before being expelled into the vacuum deposition chamber (10^{-5} mbar) through a differential pumping stage.

The metallic cluster sizes as well as the cluster deposition rate depend on the properties of target material, the DC power on the backside electrode, the argon inlet flux, the pressure in the cluster growth zone and the length of the cluster growth zone. DC power and argon flux are controlled by electronic equipment while pressure in the cluster growth is tuned by adjusting the diameter of the diaphragm at the output of the growth zone. The magnetron head is set on a translational stage enabling adjustment of the growth zone length. Hence, by proper control of the parameters, the source can produce metallic nanoclusters with tunable deposition rate and diameter (mean diameter: adjustable between 2 and 9.5 nm, depending on the target material) which is ideal for studies and calibration of our resonant mass sensors.

The setup, shown in Supplementary Figure 2a, allows for sequential acquisitions of mass deposition rates and mass spectra with TOF and NEMS-MS. The resonant device is placed on a sample stage (see

Supplementary Figure 2b). This stage is subsequently introduced in the deposition chamber of the setup. Supplementary Figure 2c shows how the setup has the ability to retract or place in the mass flux the Quartz Cristal Microbalance (QCM) and/or the NEMS. Moreover, by retracting both, a TOF mass spectrum can be acquired. We can thus easily acquire a TOF spectrum, a NEMS spectrum and the mass deposition rate with the same experimental conditions as long as the mass flux remains stable (which is half-an-hour to several hours as measured in previous studies). Thanks to deflection plates placed in the intermediate chamber (shown in Supplementary Figure 2d), we have the option to remove charged particles with an electrostatic field. A potential of 40 V removed all charged particles from the flux.

The in-line TOF spectrometer is based on a design by Wiley and McLaren[5]. It takes advantage of the fact that 10% or more of the clusters are positively ionized, eliminating the need for extra ionization. The pulsed two-stage acceleration scheme is optimized for the time-focusing of clusters on the multi-channel plate detector, located at the end of the field-free drift region. The artifact observed at 340 kDa in Figure 2 of the main text is due to light fragments produced by clusters hitting the first acceleration grid. It is systematically present during the acceleration pulse and does not correspond to clusters present in the beam. The cluster mass is deduced from the cluster time of flight through a simple set of factors: the distances between electrodes and the applied voltages. The calibration is performed in the low mass range where the atomic resolution is obtained: small clusters comprising a small number of atoms are produced, and the corresponding individual peaks are identified. In the high mass range the calibration is confirmed by comparison of TOF measurements with Transmission Electronic Microscopy (TEM) and High-Resolution TEM (HRTEM) observations[6] (see Supplementary Figure 3). For this calibration clusters are deposited on TEM grids consisting of a continuous ultrathin carbon film lying across a lacey carbon support film, supported itself by a 400 mesh copper grid. The TEM measures the projected image of particles deposited onto this electron-transparent grid. Particle size is determined by measuring the contiguous area of pixels that fall within the threshold set for a particular image. This area is then used to determine the equivalent diameter, assuming particles of spherical shape. The size histogram is built on a set of several hundreds of clusters. The procedure gives a precision of a fraction of a nanometer, although different measurement techniques of the same particles are known to yield a spread of typically 1nm[7]. Our TEM and TOF measurements are within a interval of $\pm$ 0.5 nm. As for the finite size effect on the density, it is noticeable only under a limit of about a thousand atoms, due to the fact that at this size half of the atoms lie in the outer layer with a contracted lattice parameter[8]. For clusters larger than 2 to 3 nm, one can thus safely use the bulk density for the diameter to mass conversion.

As for multiply charged clusters, while TOF spectra may present multiple peaks caused by clusters with more than one electrical charge, distributions built from TEM images always displayed one single peak. These measurements confirm that the cluster source produces a cluster distribution centered on a single peak. The additional peaks in TOF measurements always display 1/2 or 1/3 ratios with respect to the higher mass peak, indicating the presence of multi-charged particles. Regardless of multiple charging effects, the TOF provides a reliable description of the actual cluster distribution and the ionized clusters measured by the TOF are representative of the total cluster distribution.

Spectrum acquisition procedure

Both electrical and thermal controls of the NEMS were performed using specific vacuum feedthroughs providing both electrical connections and thermal contact. Cooling the device to liquid nitrogen temperature (77 K) improves the sensor frequency stability by a factor two with respect to room temperature. Once the device is under vacuum, cooled with liquid nitrogen and presents stable resonance frequencies, a multimode frequency acquisition is recorded (before the mass accretion is initiated) to characterize the frequency noise in terms of standard deviation and correlation between the two modes. The extracted noise properties are important for the data post-processing algorithm (see section below). The NEMS experimental mass sensitivity is characterized by comparing the

frequency responses of the two modes to uniform mass deposition with the deposition rate obtained with the QCM (see section below “mass sensitivity calibration”). The mass deposition rate and a TOF mass spectrum of the nanoclusters flux are acquired before and after each NEMS exposure to the nanoclusters flux to ensure that the particle population remains the same during the NEMS-MS acquisition. Multimode frequency jumps are recorded for a few minutes yielding thousands of detected events. Frequency jumps are subsequently detected automatically, quantified and a mass spectrum is generated. Jump detection relies on noise subtraction and a Student t-test function (see section below) while the spectrum construction models the probability density function associated with each mass landing event as detailed elsewhere[4].

Jump detection algorithm

We developed a simple but robust step detection algorithm based on two steps: data denoising and precise step localization with the Student’s t-test. Denoising the data reveals abrupt frequency jumps on which the Student’s t-test is applied to localize its center.

As mentioned in the main text, denoising requires a preliminary step consisting in acquiring noise data in multimode operation: the resonance frequencies of mode 1 and mode 2 are simultaneously monitored under typical operating conditions in the absence of any mass landing events. The frequency raw data points are averaged over some integration time τ_{avg} before computation of the fractional frequency differences:

$$y_n(i) = \frac{f_n(i+1) - f_n(i)}{f_n} \quad \textbf{(1)}$$

n = 1 or 2 for mode 1 or 2 respectively y_n denotes the relative frequency change from one point to the next. τ_{avg} is usually a multiple of τ_{PLL}, typically $3\tau_{PLL}$.

A scatter plot of fractional frequency differences of mode 2 with respect to mode 1 can be fitted with a bivariate Gaussian distribution defined by three parameters σ1, σ2 and ρ, respectively the frequency standard deviation of mode1, mode 2 and their correlation factor. Hence, the resonator noise can be modeled by a Joint Probability Density Function (JPDF)[4] (µ1 and µ2 being the characterized mean frequency fluctuations of the two modes).

$$\mathrm{JPDF}_{\delta f_1,\delta f_2}(\delta f_1, \delta f_2) = \frac{1}{2\pi\sigma_1\sigma_2\sqrt{1-\rho^2}} e^{-\frac{z}{2(1-\rho^2)}}$$

where

$$z = \frac{(\delta f_1 - \mu_1)^2}{\sigma_1^2} - \frac{2\rho(\delta f_1 - \mu_1)(\delta f_2 - \mu_2)}{\sigma_1\sigma_2} + \frac{(\delta f_2 - \mu_2)^2}{\sigma_2^2} \quad \textbf{(2)}$$

These parameters can be easily fitted and used to obtain an elliptical contour fit calculated at a chosen multiple of σ_1 and σ_2 as shown in Supplementary Figure 4a. The extracted standard error ellipse can then be used as a “denoising” tool on data acquired during a mass adsorption experiment: fractional

frequency differences of mode 2 are plotted with respect to those of mode 1. Points lying outside of the standard error ellipse are considered as mass adsorption events. Points inside the ellipse are attributed to the NEMS intrinsic noise and discarded. For typical experimental conditions, a 5σ ellipse discards most noise-induced fractional frequency shifts and long term drifts. An example of denoising with a 5σ standard error is shown Supplementary Figure 4a. The denoised data shows plateaus and abrupt jumps that provide a list of frequency change-point timestamps as shown in Supplementary Figure 4b. In a very short range centered on each timestamp, a Student's t-test[9] is applied on the raw frequency data. The maximums of the t functions computed over the chosen range provide us with a good estimation of the center coordinates of the frequency jumps (see Supplementary Figure 4c).

For each jump, the resonator frequency is averaged over τ_{avg} before and after the jump. The frequency shift magnitude is measured as illustrated in Supplementary Figure 4d. Events where a frequency shift is observed on both modes form pairs of frequency shifts $[\delta f_1, \delta f_2]$ that can be translated to mass/position pairs[4] $[\delta m, x]$ where $\delta m = \frac{m}{M}$ is the particle mass relative to the total mass of the resonator M and x the relative position of the particle along the beam. Using a multimode analysis described elsewhere[4], each mass/position pair $[\delta m, x]$ is associated to its measurement error via a probability density function (PDF). An event is then characterized by its (most probable) central mass δm as well as its probability of occurrence like shown in Supplementary Figure 4e. Finally, the final spectrum is constructed in real time, event per event, by summing all these events (see Supplementary Figure 4b).

TOF-MS and NEMS-MS spectra

Many different spectra were acquired and constructed with both NEMS and TOF, over several weeks with different NEMS devices. Parameters were varied (see section above "Nanocluster growth and detection") to obtain various cluster population in mass and size, and even materials (copper and tantalum). See three examples shown Supplementary Figure 5. Good reproducibility and stability were found for all these measurements.

Mass sensitivity calibration

The nanoresonator mass sensitivity can be calibrated by comparing its frequency response to uniform mass deposition with the mass deposition rate provided by the QCM.

Mass landings on the resonant beam induce frequency shift pairs $[\delta f_1, \delta f_1]$ that can be translated to pairs $[\delta m, x]$, with $\delta m = \frac{m}{M}$. Thus precise determination of the total mass M of the resonator is essential for accurate estimation of the particle masses. However, while the nominal resonance frequencies f_1, f_2 or the mass-induced frequency shifts $[\delta f_1, \delta f_2]$ can be obtained experimentally, we have no direct measurement of M. We propose the experimental determination of this value by characterizing the linear frequency response to uniform mass deposition along the beam. The theoretical frequency response of a beam resonator to a uniform deposition along its length leading to a total added mass $\Delta m \ll M$ is

$$\frac{\Delta f_n}{f_n} = \frac{1}{2}\frac{\Delta m}{M} \quad \textbf{(3)}$$

where f_n is the resonance frequency of mode n and M is the total mass of the resonator.

The QCM is used to measure the mass rate per unit area of the particle flow and the total mass deposited on the sensor in a given time Δt is:

$$\Delta m = \varphi_{QCM}.L.w.\Delta t \qquad \textbf{(4)}$$

Where φ_{QCM} is the deposition rate measured in $kg.s^{-1}.m^{-2}$, L the beam length and w the beam width. Finally the resonator mass is obtained by combining 3 and 4:

$$M = \frac{1}{2}\frac{f_n}{\left(\frac{\Delta f_n/\Delta t}{\varphi_{QCM}Lw}\right)} \qquad \textbf{(5)}$$

L and w are precisely measured by SEM observation (The resonator length is measured between the two nanogauge bridges. Indeed, FEM simulations showed that the portion of the beam comprised between the anchors and the nanogauges underwent negligible displacement). SEM images confirmed the 300 nm width of our resonators. The mass sensitivity of the resonator $\frac{\Delta f_n/\Delta t}{\varphi_{QCM}Lw}$ is the slope of a graph showing the rate of frequency change versus the mass deposition rate measured by the QCM. Finally if one writes $M = \rho_{Si}tLW$, Supplementary Equation (5) provides an experimental determination of $\rho_{Si}t$ (the thickness being the main unknown here due to silicon top layer grinding).

For this calibration procedure, the focus is not on frequency jumps caused by individual events but rather on the average slope of the frequency drifts during mass deposition over sufficiently long time ranges. Low cluster diameters and high flux rates are used to ensure uniform mass deposition along the beam (see Supplementary Figure 6). Different mass deposition rates are obtained by tuning the parameters of the cluster source while monitoring the result with the QCM before frequency shift acquisition on the NEMS. Then the NEMS frequency shift rate is extracted from each acquisition and compared to the QCM. A linear fit yields the experimental mass sensitivities. The method is illustrated in Supplementary Figure 7 for a 9.835µm long beam with tantalum clusters deposition.

In this particular case, the obtained mode 1 and mode 2 experimental sensitivities are 14.6 $Hz.ag^{-1}$ and 40.1 $Hz.ag^{-1}$ respectively. The measured experimental mass is found to be equal to 0.89 pg from (5) (the same value is consistently obtained for both modes). This value is slightly smaller than the calculated value from the theoretical resonator dimensions: the theoretical mass is equal to 1.01 pg. The experimental mass was generally found from 0 to 15% smaller than the theoretical resonant mass. We attributed this discrepancy to device thickness variations on the different wafers (which is consistent with manufacturer's data sheet) or to uncertainties on the boundary conditions caused by the time-defined vapor HF release underetch.

Supplementary References

1. Kharrat, C., Colinet, E. & Voda, A. H-infinity Loop shaping control for PLL-based mechanical resonance tracking in NEMS resonant mass sensors. in *IEEE Sensors, 26-29 Oct. 2008* 1135 – 1138 (2008).

2. Mile, E. *et al.* In-plane nanoelectromechanical resonators based on silicon nanowire piezoresistive detection. *Nanotechnology* **21,** 165504 (2010).

3. Bargatin, I., Myers, E. B., Arlett, J., Gudlewski, B. & Roukes, M. L. Sensitive detection of nanomechanical motion using piezoresistive signal downmixing. *Appl. Phys. Lett.* **86,** 133109 (2005).

4. Hanay, M. S. *et al.* Single-protein nanomechanical mass spectrometry in real time. *Nat. Nanotechnol.* **7,** 602–608 (2012).

5. Wiley, W. C. & McLaren, I. H. Time-of-Flight Mass Spectrometer with Improved Resolution. *Rev. Sci. Instrum.* **26,** 1150 (1955).

6. Morel, R., Brenac, A., Portemont, C., Deutsch, T. & Notin, L. Magnetic anisotropy in icosahedral cobalt clusters. *J. Magn. Magn. Mater.* **308,** 296–304 (2007).

7. Small, J. A. Gold nanoparticles, nominal 10nm diameter. *NIST Rep. Investig.* (2012).

8. Tomanek, D., Mukherjee, S. & Bennemann, K. H. Simple theory for the electronic and atomic structure of small clusters. *Phys. Rev. B* **28,** 665–673 (1983).

9. Carter, N. J. & Cross, R. a. Mechanics of the kinesin step. *Nature* **435,** 308–12 (2005).
